# Supplementary material for: Ruxolitinib for the treatment of acute graft-versus-host disease: a retrospective analysis
Source: Ann Hematol. 2024 Jun 25;103(8):3071–81. doi: 10.1007/s00277-024-05696-x (PMC11283387; doi:10.1007/s00277-024-05696-x)
Supplement: Supplementary file 1 — Supplementary Material 1 [file 277_2024_5696_MOESM1_ESM.doc]

**Supplementary material**

**Table S1 Response assessment depending on MAP score (MAP 1 vs MAP 2+3)**

|  | **Responder*** | **Non-responder** | **p-value** |
| --- | --- | --- | --- |
| **2BM Ann Arbor Score (AA1), onset GvHD (n, %)** | 5 (83) | 1 (17) | 0.63° |
| **2BM Ann Arbor Score (AA2+AA3), onset GvHD (n, %)** | 15 (63) | 9 (27) |
| **2BM Ann Arbor Score (AA1), start ruxolitinib (n, %)** | 4 (80) | 1 (20) | 0.63° |
| **2BM Ann Arbor Score (AA2+AA3), start ruxolitinib (n, %)** | 19 (58) | 14 (42) |
| *defined as CR or PR after one month  ° Fisher’s exact test was performed.  BM= biomarker; GvHD=acute graft-versus-host disease. | | | |

**Table S2 FFS depending on** single MAP scores

|  | **FFS** | | |
| --- | --- | --- | --- |
|  | **MAP1** | **MAP2** | **MAP3** |
| **1 week (n, %)** | 5/5 (100%) | 17/17 (100%) | 15/16 (94%) |
| **1 month (n, %)** | 4/5 (80%) | 14/17 (82%) | 10/16 (63%) |
| **3 months (n, %)** | 4/5 (80%) | 8/16 (50%) | 7/15 (47%) |
| **6 months (n, %)** | 3/4 (75%) | 7/16 (44%) | 4/13 (31%) |
| FFS=failure free survival; MAP= MAGIC Algorithm Probability | | | |

FFS was compared between patients with MAP 1 (n=5) vs MAP 2+3 (n=33). FFS was 100% (5/5 patients) after one week, 80% (4/5) after 1 month, 80% (4/5) after 3 months, and 75% (3/4) after 6 months for MAP 1, while FFS was 97% (32/33 patients) after 1 week, 73% (24/33) after 1 month, 48% (15/31) after 3 months, and 38% (11/29) after 6 months for MAP 2+3.
