# Supplementary material for: Ruxolitinib for the treatment of acute graft-versus-host disease: a retrospective analysis
Source: Ann Hematol. 2024 Jun 25;103(8):3071–81. doi: 10.1007/s00277-024-05696-x (PMC11283387; doi:10.1007/s00277-024-05696-x)
Supplement: Supplementary file 2 — Supplementary Material 2 [file 277_2024_5696_MOESM2_ESM.doc]

**Title**

Ruxolitinib for the treatment of acute graft-versus-host disease: a retrospective analysis

**Authors**

Alexander Denk^1^, Matthias Edinger^1^, Daniela Weber^1^, Ernst Holler^1^, Matthias Fante^1^, Elisabeth Meedt^1^, Sibel Gunes^2^, Hendrik Poeck^1^, Cornelia Mittermaier^1^, Wolfgang Herr^1^, Daniel Wolff^1^

**Affiliations**

^1^ Dept. of Internal Medicine III, Hematology & Oncology, University Hospital Regensburg, Germany

^2^ Novartis Pharma AG, Basel, Switzerland

**Correspondence to**

Daniel Wolff, University Hospital Regensburg

Department of Internal Medicine III

Franz-Josef-Strauss-Allee 11, 93053 Regensburg, Germany

Phone: +49 / 941 / 944-15531

Fax: +49 / 941 / 944-81-15542

e-mail: daniel.wolff@ukr.de

Acknowledgement: Editorial support under the guidance of the authors was provided by Anupama Tamta, of Novartis Healthcare Pvt Ltd, Hyderabad in accordance with Good Publication Practice (GPP 2022) guidelines (http:www.ismpp.org/gpp-2022).

**Abstract**

Steroid-refractory acute graft-versus-host disease (aGvHD) is a serious complication after allogeneic hematopoietic stem cell transplantation, associated with significant mortality. Ruxolitinib was the first drug approved for aGvHD, based on results of the REACH2 trial; however, real-world data are limited. We retrospectively analyzed the safety and efficacy of ruxolitinib for treatment of aGvHD at our center from March 2016 to August 2022 and assessed biomarkers of risk. We identified 49 patients receiving ruxolitinib as second- (33/49), third- (11/49), fourth- (3/49), or fifth-line (2/49) treatment. Ruxolitinib was started on median day 11 (range, 7–21) after aGvHD onset; median duration of administration was 37 days (range, 20–86), with 10 patients continuing treatment at last follow-up. Median follow-up period was 501 days (range, 95–905). In the primary analysis at the 1-month assessment, overall response rate was 65%, and failure-free survival was 78%. Infectious complications ≥CTCAE Grade III were observed in 10/49 patients within 1-month follow‑up. Patients responding to ruxolitinib therapy required fewer steroids and exhibited lower levels of the serum biomarkers regenerating islet-derived protein 3-alpha, suppression of tumorigenicity 2, and the Mount Sinai Acute GVHD International Consortium algorithm probability. A univariate regression model revealed steroid-dependent aGvHD as a significant predictor of better response to ruxolitinib. Within 6-months follow-up, four patients experienced recurrence of underlying malignancy, and eight died due to treatment-related mortality. Overall, ruxolitinib was well‑tolerated and showed response in heavily pretreated patients, with results comparable to those of the REACH2 trial. Biomarkers may be useful predictors of response to ruxolitinib.

**Keywords**

Acute graft-versus-host disease, aGvHD, allogeneic hematopoietic stem cell transplantation, ruxolitinib, steroid‑refractory acute graft-versus-host disease, JAK1/2 kinase inhibitor

**Introduction**

Acute graft-versus-host disease (aGvHD) is a major complication that occurs in approximately 50% of patients after allogeneic hematopoietic stem cell transplantation (allo-HSCT) and significantly contributes to nonrelapse mortality and a reduced quality of life [1–6]. Glucocorticoids (GCs) combined with calcineurin inhibitors (CNIs) represent the backbone of aGvHD treatment [7]. However, a significant proportion of patients lack sustained response to GCs [8,9]. Currently, no standard second-line treatment has been established for aGvHD, and treatment depends on center-specific preferences. The recent approval of ruxolitinib for the treatment of steroid-refractory aGvHD may provide an option for a standardized treatment for this condition. Based on increasing knowledge on the pathogenesis of aGvHD [1,10–12], including the role of the JAK/STAT signaling pathway in immune cell activation and tissue inflammation during GvHD, ruxolitinib, an oral JAK1/2 kinase inhibitor, was explored in its treatment [13,14]. Based on promising results in the controlled, randomized REACH2 and 3 trials [15,16], ruxolitinib was approved in 2019 for the second-line treatment of aGvHD, and later for chronic GvHD (cGvHD), by the Food and Drug Administration (FDA) and the European Medicines Agency (EMA) [17–19]. Since the REACH2 trial was conducted on selected patients in a second-line treatment setting, we retrospectively analyzed the efficacy and safety in all (unselected) patients receiving ruxolitinib for the treatment of aGvHD, including those receiving multiple treatment lines, between 2016 and 2022 at the University Hospital of Regensburg. This analysis was combined with the assessment of established biomarkers for aGvHD [20–22] (regenerating islet-derived protein 3-alpha [REG3α], suppression of tumorigenicity 2 [ST2], and the derived Mount Sinai Acute GVHD International Consortium (MAGIC) algorithm probability [MAP]) to further characterize the treated patient population with regard to the risk profile for treatment-related mortality.

**Patients and methods**

**Patients**

All 49 patients treated with ruxolitinib for aGvHD between March 2016 and August 2022 at the University Hospital Regensburg, Germany, were included in this retrospective analysis, which was approved by the institutional review board (no. 22-3076-104) and performed in compliance with the current Declaration of Helsinki. All cases were analyzed and pseudonymized, and living patients provided written informed consent for publication. The diagnosis, assessment of organ involvement, and documentation of aGvHD were performed as part of routine clinical practice, either during inpatient therapy or follow-up outpatient visits. Criteria established by Glucksberg and Thomas, which were recently updated by the Mount Sinai Acute GvHD International Consortium (MAGIC), were used in these assessments [23–26], either in the context of inpatient therapy or during follow-up outpatient visits.

**Definition of response to ruxolitinib treatment and adverse events**

Clinical response was evaluated at 1 week, 1 month, 3 months, and 6 months after the start of ruxolitinib therapy. The aGvHD grading, recently updated by the MAGIC consortium, and the intensity of immunosuppression (IS) were assessed at the start of ruxolitinib treatment and repeated after 1 week, 1 month, 3 months, and 6 months. Response assessment was terminated at the start of any additional new immunosuppressive medication (ISM). Complete remission (CR) was defined as the resolution of all symptoms of aGvHD without starting any new additional ISM while receiving ruxolitinib treatment. Partial response (PR) was defined as an improvement of at least one organ grade without the progression of aGvHD to other organs. Mixed response (MR) was defined as an improvement (at least PR) in one organ, with concurrent progression in another organ site. Progressive disease (PD) was defined as disease progression in at least one organ without any improvement in other organ sites. Stable disease (SD) was defined as stable organ involvement without any changes in grading. For the evaluation of predictive markers, patients were divided into two groups at 1-month follow-up: “responders” (CR and PR) and “nonresponders” (MR, SD, PD, and additional ISM); At the time of 1-month follow-up, three patients had already died. For the latter patients, the last response assessment was used. Failure-free survival (FFS) was defined as the absence of relapse or nonrelapse mortality without the administration of further ISM. Overall response rates (ORRs) were calculated based on an intention-to-treat analysis. Durable ORR (assessed at months 3 and 6) was defined as the proportion of patients who maintained a response (CR or PR) since month 1. Infectious complications and hematological toxicities were assessed according to the Common Terminology Criteria for Adverse Events version 5.0 (CTCAE 5.0).

**Statistical analyses**

Analyses were performed using absolute and percentage frequency (n and %) and median with interquartile range (IQR). Due to the limited number of patients, univariate analyses were conducted. The effects of 14 clinical and demographic parameters (age, weight, sex, time to start of ruxolitinib after onset of aGvHD, initial ruxolitinib dose, severity of aGvHD, additional ISM, treatment lines before ruxolitinib, affected organ site, response to steroids, CTCAE anemia, CTCAE thrombocytopenia, CTCAE neutropenia and MAP at start of ruxolitinib) on response to ruxolitinib treatment were analyzed using univariate binary logistic regressions. Odds ratios (ORs) with 95% confidence intervals (CIs), are presented. Statistical analyses were conducted using IBM SPSS Statistics, version 26 (IBM Corp., Armonk, NY, USA). The level of significance was set at a two-sided p-value of ≤0.050. The GC-sparing effect during ruxolitinib treatment was assessed using nonparametric matched pairs analysis (Wilcoxon signed-rank test). Comparison of cytopenia at the start of treatment and within 1 month after ruxolitinib treatment (paired nominal data) was conducted using nonparametric McNemar test; Assessment of severe adverse events of cytopenia was also conducted using the nonparametric McNemar test.

**Measurement of Reg3α and ST2 in the serum**

Reg3α and ST2 serum concentrations were measured using enzyme-linked immunosorbent assay, as previously described, and are reported in nanograms per milliliter (ng/mL) and picograms per milliliter (pg/mL), respectively [20,27,28]. MAP, which combines the serum concentrations of both biomarkers, was analyzed based on studies from the Mount Sinai Acute GvHD International Consortium (MAGIC [29]. Serum Reg3α and ST2 were sampled at (i) the onset of aGvHD and (ii) the start of ruxolitinib treatment. For ruxolitinib treatment, samples taken within a timeframe of 7 days before and 2 days after the first dose of ruxolitinib were considered. The Mann-Whitney-U-test was used to compare Reg3α and ST2 serum concentrations between responders and nonresponders.

Differences in MAP risk classification between responders and nonresponders at (i) the onset of aGvHD and (ii) the start of ruxolitinib treatment were evaluated using the Chi-squared test.

**Results**

**Patient characteristics**

A total of 49 patients (male, n=29; female, n=20) treated with ruxolitinib for aGvHD between March 2016 and August 2022 were included in this analysis. Details of the patient characteristics and ruxolitinib treatment are presented in Tables 1 and 2. The median age at the time of allo-HSCT was 55 years (range, 46–61). In total, 39 patients received a donor graft from unrelated donors, and 10 received grafts from related donors. In two cases, aGvHD occurred after donor lymphocyte infusion (DLI) following grafting from unrelated donors.

GvHD prophylaxis included cyclosporin A (CsA) plus methotrexate (MTX) in 30 patients, cyclophosphamide/tacrolimus/mycophenolate mofetil (MMF) in 10, tacrolimus plus MMF in five, and CsA plus MMF in two; CsA plus bortezomib and cyclophosphamide/everolimus/MMF were each used in one patient. 33 patients received additional antithymocyte globulin (ATG) prophylaxis. The onset of aGvHD occurred on median day 20 (range, 16–27 days). Ruxolitinib was started on median day 59 (range, 41–97 days) after allo-HSCT and on median day 11 (range, 7–20) after the onset of the aGvHD episode leading to ruxolitinib therapy. Before the start of ruxolitinib treatment, the maximum severity of aGvHD was Grade II and III+IV in 39% and 53% of the patients, respectively. At the start of ruxolitinib treatment, aGvHD was Grade II and III+IV in 45% and 41% of the patients, respectively, whereas 14% of the patients received ruxolitinib for persistent skin aGvHD stage II (overall Grade I). In most patients, the main manifestation of aGvHD was in the gut (61%), followed by the skin (37%) and liver (2%). The median line of ruxolitinib therapy was second-line (range, 2–3), with 33 patients (67%) receiving ruxolitinib as second-line, 11 patients (22%) as third-line, three patients (6%) as fourth-line, and two patients (4%) as fifth-line treatment.

The median duration of ruxolitinib treatment was 37 days (range, 20–86), and the median follow-up period after assessment was 501 days. At the last follow-up, 11 patients were receiving ongoing therapy. In addition to ruxolitinib, 23 patients (47%) received two additional ISMs, 20 (41%) received three additional ISMs, five (10%) received four additional ISMs, and one (2%) received one additional ISM. The most common combination was prednisolone/CNI/ruxolitinib (45%). The median GC (prednisolone) dose at the start of ruxolitinib treatment was 1.3 mg/kg (range, 0.6–1.9 mg/kg). Fifteen patients (31%) were diagnosed with steroid‑dependent aGvHD, and 34 (69%) with steroid-refractory aGvHD. All patients received an antifungal prophylaxis with a mold active agent with posaconazole used first-line and in case of breakthrough infections isavuconazole. The use of concomitant azoles was not considered in dosing of ruxolitinib.

**Response to ruxolitinib**

***Response to ruxolitinib at 1 week***

One week after starting ruxolitinib therapy, six patients (12%) achieved CR, 19 (39%) achieved PR, one (2%) achieved an MR, 18 (37%) had SD, and four (8%) had PD. Additional ISM was initiated in one patient (2%). The ORR was 51% (25/49), and FFS was 98% (48/49).

***Response to ruxolitinib at 1 month***

One month after the first administration of ruxolitinib, 24 patients (49%) achieved CR and eight (16%) achieved PR. Of those patients with PR, one patient who had prior CR suffered from a flare of aGvHD 14 days after ruxolitinib therapy (which had been administered for 18 days) was discontinued and achieved PR with prednisolone treatment alone. One patient (2%) achieved an MR, and four (8%) had SD. In one of the patients with stable GvHD, ruxolitinib was terminated due to hematotoxicity without the addition of a new ISM. One patient (2%) had PD; therefore, ruxolitinib administration was discontinued. New ISMs were administered to seven patients. Among these, one patient experienced a relapse of AML. Overall, three patients (one with PD and two with SD) died due to aGvHD of the gut (n=1), aGvHD of the skin and gut complicated by sepsis (n=1), or Pseudomonas pneumonia (n=1). The ORR was 65% (32/49), and FFS was 78% (38/49).

***Response to ruxolitinib at 3 months***

Three months after the start of ruxolitinib therapy, 23 patients (53%) achieved CR, two (5%) achieved PR, and one (2%) had PD. Since the last follow-up, two additional patients (5%) experienced a relapse of aGvHD: one patient required a new ISM, and the other patient had a relapse of aGvHD after the termination of ruxolitinib due to sepsis and then received ruxolitinib (+etanercept) treatment. Three more patients (7%) started additional ISM, and one patient died due to late-onset aGvHD of the gut. Another patient in whom ruxolitinib treatment had been discontinued at the 1-month follow-up died. Two of the aforementioned patients (one with CR and one who received a new ISM) also experienced a relapse of hematologic malignancy. Of note, two patients developed cGvHD but did not receive additional systemic ISM. At the end of the study period, six patients had not yet reached the 3-month follow-up period and were excluded from the ORR and FFS calculations. The ORR was 58% (25/43), durable ORR (1m/3m) was 53% (23/43), and FFS was 58% (25/43).

***Response to ruxolitinib at 6 months***

At 6 months, 19 patients (50%) achieved a CR. Since the last follow-up, three patients received a new ISM due to cGvHD and two (5%) died (TRM). Another patient, who had received an additional ISM after three months, died due to an epidural hematoma (TRM). Of note, seven of the prior mentioned patients with CR developed cGvHD not requiring a new ISM. In total, eight patients receiving ongoing ruxolitinib therapy had not yet reached the 6‑month follow‑up and were excluded from the ORR and FFS calculations as were those who received new ISM due to cGvHD. The ORR was 50% (19/38), with a durable ORR of 45% (17/38), and FFS of 47% (18/38). The ORR and FFS are graphically presented in Fig. 1. In total, during the 6-month follow-up period, four patients experienced a relapse of hematologic malignancy, and eight patients died.

**Safety: infectious adverse events and other complications during ruxolitinib therapy within 1 month of treatment initiation**

At the start of ruxolitinib therapy, 42 patients (86%) had anemia, including 21 (43%) with anemia ≥CTCAE Grade III; 44 patients (90%) had thrombocytopenia, including 24 (49%) with thrombocytopenia ≥CTCAE Grade III; and 10 patients (20%) had neutropenia, including five (10%) with neutropenia ≥CTCAE Grade III. Within one month after the start of ruxolitinib therapy, 42 patients had anemia; however, the proportion of patients with anemia ≥CTCAE Grade III increased (29 patients [59%], p=0.057). Additionally, all patients had thrombocytopenia of any grade (p=0.063) and 19 patients (39%) had neutropenia (p=0.035). Overall, 32 patients (65%) had thrombocytopenia ≥CTCAE Grade III (p=0.057), and 14 patients (29%) had neutropenia ≥CTCAE Grade III (p=0.033).

52 events of infections occurred in 31 patients (63%) within the first month after treatment initiation, including 14 events of ≥CTCAE Grade III in 10 patients (20%). Infectious adverse events ≥CTCAE Grade III included cytomegalovirus reactivation with a need for systemic therapy (n=4), BK-virus-cystitis (n=3), Epstein-Barr virus reactivation requiring systemic therapy (n=1), *Klebsiella pneumoniae*–associated urosepsis (n=1), *Staphylococcus aureus*–associated sepsis (n=1), sepsis with an unknown pathogen (n=1), Pseudomonas pneumonia with aspergillosis of the gut (n=1), and fungal pneumonia (n=1; Table 3).

**Steroid-sparing effect of ruxolitinib**

As shown in Fig. 2, the median steroid dose in all patients who completed the follow-up decreased over the course of the follow-up period. After 1 week, the median steroid dose had already reduced significantly from 1.25 mg/kg (IQR 0.55–1.93 mg/kg; n=49) to 1.07 mg/kg (IQR 0.49–1.71 mg/kg; n=48; p≤0.001). At 1 month (n=38), 3 months (n=24), and 6 months (n=12) after the start of ruxolitinib treatment, the median steroid dose was 0.37 mg/kg (IQR 0.24–0.70 mg/kg; p≤0.001), 0.12 mg/kg (IQR 0.08–0.20 mg/kg; p≤0.001), and 0.04 mg/kg (IQR 0–0.08 mg/kg; p=0.002), respectively. A comparison between the median steroid doses after 1 week and 1 month confirmed a significant decrease (p≤0.001; Fig. 1). After 1 month, responders (n=32) received a median steroid dose of 0.33 mg/kg (IQR 0.23–0.64 mg/kg), while nonresponders (n=6) received 0.82 mg/kg (IQR 0.43–2.04 mg/kg; p=0.045).

**Factors associated with response to ruxolitinib** **treatment**

In total, 14 factors were analyzed to assess if there was an association with response to ruxolitinib treatment. Of these, two factors, need for additional ISM and response to steroids, showed statistically significant associations.

Patients who received fewer ISMs in addition to ruxolitinib at the start of therapy showed a statistically better response to ruxolitinib (p=0.006). Patients with steroid-dependent aGvHD also showed a significantly better response to ruxolitinib (p=0.021; Tables 4 and 5).

As shown in Table 6, plasma concentrations of REG3α and ST2 were higher in patients who failed to respond to ruxolitinib after 1 month, both at the onset of aGvHD and the start of ruxolitinib treatment. Additionally, patients responding to treatment had lower MAP scores both at the onset of aGvHD and the start of ruxolitinib therapy. In patients with low (Ann Arbor 1, MAP<0.141) or intermediate (Ann Arbor 2, 0.141≤MAP≤0.290) MAP scores at the onset of GvHD or the start of ruxolitinib treatment, the response was better than that in patients with high initial MAP scores (Ann Arbor 3, MAP>0.290). FFS based on the MAP scores was also analyzed. Interestingly, a comparatively small number of patients (n=5) presented with MAP 1 at the start of ruxolitinib treatment, most likely reflecting the fact that the majority of this patient cohort had moderate and severe aGvHD with a high proportion of steroid-refractory aGvHD. In contrast, 17 and 16 patients presented with MAP 2 and 3, respectively. Therefore, FFS was compared between patients with MAP 1+2 (n=22) and those with MAP 3 (n=16). In line with the aforementioned results, patients with lower MAP scores had better FFS. FFS was 100% (22/22 patients) after 1 week, 82% (18/22) after 1 month, 57% (12/21) after 3 months, and 50% (10/20) after 6 months for MAP 1+2, whereas it was 94% (15/16 patients) after 1 week, 63% (10/16) after 1 month, 47% (7/15) after 3 months, and 31% (4/13) after 6 months for MAP 3 (Fig. 3). FFS analysis depending on a single MAP score (MAP 1/2/3) and response assessment in terms of conventional classification (MAP 1 vs MAP 2+3) is presented in the supplementary tables S1 and S2, respectively.

**Discussion**

With an incidence of 30–60% and a mortality rate of 15–30%, aGvHD is a major complication after allo-HSCT [30–32]. According to the European Group for Blood and Marrow Transplantation (EBMT) guidelines, the first‑line treatment for aGvHD Grade II–IV is systemic GC [7]. Unfortunately, approximately 60–70% of patients with severe aGvHD and 40% of patients with mild or moderate aGvHD do not respond to systemic GC or experience relapse [6,33,34]. To date, no standard second-line treatment has been established [7,35], with ruxolitinib being the only drug approved for the treatment of steroid-refractory GvHD based on the results of the randomized REACH2 trial [15,36,37] evaluating ruxolitinib as a second-line treatment.

While in our retrospective cohort, ruxolitinib was administered as a second-line treatment to 33 patients (67%), a significant proportion of patients received ruxolitinib as third- (22%), fourth- (6%), or fifth-line (4%) treatment. The ORR after 1 month of ruxolitinib therapy was 65%, which is comparable to the results of the REACH1 trial and the randomized REACH2 trial (ORR after 28 days: 55% and 62%, respectively) [15,37]. The slightly higher ORR reported in our study might be because some patients were treated for residual aGvHD Grade I and fewer patients had high-grade aGvHD (i.e., Grade III–IV; 41% vs 68% in the REACH1 trial). In the REACH2 trial, FFS was 82% after 1 month and 47% after 6 months, which is also comparable to our findings (78% and 47%, respectively).

In line with the results from the REACH1 and 2 trials, responses were observed regardless of organ involvement, with the skin and gastrointestinal tract representing the most frequently affected organs (p=0.79). As in our study only one patient received ruxolitinib predominantly for liver GvHD, no valid conclusions could be drawn in this regard.

The most frequently reported side effects of ruxolitinib therapy are infectious complications and cytopenia. Given that ruxolitinib was started at a median of 59 days after allo-HSCT, it is not surprising that many patients in our cohort already displayed anemia (86%), thrombocytopenia (90%), and neutropenia (20%) at the onset of therapy. In contrast, after 1 month, all patients had thrombocytopenia, and 39% had neutropenia, indicating a significant increase of neutropenia due to ruxolitinib toxicity and a nonsignificant increase of thrombocytopenia. However, after 1 month, 86% of the patients had anemia. In terms of cytopenia ≥ CTCAE Grade III, there was a significant increase in neutropenia within the first month after the onset of therapy and a nonsignificant increase in anemia and thrombocytopenia.

While ruxolitinib has been associated with infectious complications in myeloproliferative disorders [38,39], data on infectious complications after allo-HSCT are limited due to presence of multiple risk factors in the latter patient cohort. However, in the context of aGvHD, infections are a common complication [15,40]. In line with this, 63% of the patients in our cohort developed infectious complications within the first month of treatment, including 20% with ≥CTCAE Grade III events.

In our analysis, we found a significantly better response rate in patients with steroid-dependent aGvHD compared with patients with steroid-refractory GvHD. Moreover, patients who received fewer additional ISMs responded significantly better to ruxolitinib treatment.

Of note, patients not responding to ruxolitib therapy had higher levels of Reg3α and ST2 in the serum both at the onset of aGvHD and the start of ruxolitinib treatment which is in line with prior publications [20,21]. Therefore, our findings suggest that these biomarkers could potentially correlate with the response to ruxolitinib treatment and, therefore, may predict severe, ruxolitinib-resistant aGvHD, with a consecutive need for additional therapeutic targeting. However, given the limited number of patients and the fact that our results were not of statistical significance, further studies in this regard are warranted.

In the context of cGvHD, a steroid-sparing effect of ruxolitinib has been described [40,41]. In our analysis, a meaningful reduction in the steroid dose for patients with aGvHD was confirmed from the first week onward, and responders had significantly lower prednisolone requirements after 1 month of ruxolitinib treatment (p=0.045).

In conclusion, ruxolitinib is an important treatment option for patients with aGvHD and is associated with steroid-sparing activity. Relevant side effects include cytopenia and infectious complications, which should be closely monitored during therapy.

**Author contributions**

Alexander Denk: data collection, analysis of the data, writing of the manuscript

Matthias Edinger: treatment of patients, correction of the manuscript

Daniela Weber: treatment of patients, supervision of MAP assessment, correction of manuscript

Ernst Holler: supervision of MAP assessment, treatment of patients, study conception (MAP)

Matthias Fante: treatment of patients, correction of the manuscript

Elisabeth Meedt: MAP assessment, correction of the manuscript, treatment of patients

Sibel Gunes: support of the research project, study conception, correction of the manuscript

Hendrik Poeck: treatment of patients, correction of the manuscript

Cornelia Mittermaier: treatment of patients, correction of the manuscript, data collection

Wolfgang Herr: supervision of the project, correction of the manuscript

Daniel Wolff: study conception, data collection, correction of manuscript

**Declarations**

All procedures performed in studies involving human participants were in accordance with the ethical standards of the responsible institution and/or national research committee and with the 1964 Helsinki Declaration and its later amendments or comparable ethical standards. Informed consent was obtained from all patients for being included in the study.

**Funding**

Daniel Wolff has received a research grant from Novartis. This study was supported by Novartis Pharma AG, Basel, Switzerland, as a part of a research collaboration. Elisabeth Meedt holds a fellowship by Else-Kröner-Fresenius-Stiftung.

**Conflicts of interest**

Daniel Wolff received honoraria from Mallinckrodt, Neovi, Takeda, Sanofi, Incyte and Novartis. Sibel Güneş is an employee of Novartis Pharma AG. All other authors have no competing interests.

**Data Availability**

The data that support the findings of this study are available from the corresponding author upon reasonable request.

**References**

1. Jagasia M, Zeiser R, Arbushites M, Delaite P, Gadbaw B, Bubnoff NV (2018) Ruxolitinib for the treatment of patients with steroid-refractory GvHD: an introduction to the REACH trials. Immunotherapy 10::391–402. https://doi.org/10.2217/imt-2017-0156
2. Martin PJ, Rizzo JD, Wingard JR et al (2012) First- and second-line systemic treatment of acute graft-versus-host disease: recommendations of the American Society of Blood and Marrow Transplantation. Biol Blood Marrow Transplant 18:1150–1163. https://doi.org/10.1016/j.bbmt.2012.04.005
3. Zeiser R, Blazar BR (2017) Acute Graft-versus-Host Disease - Biologic Process, Prevention, and Therapy. N Engl J Med 377:2167–2179. doi:10.1056/NEJMra1609337
4. van Groningen LF, Liefferink AM, de Haan AF, et al (2016) Combination Therapy with Inolimomab and Etanercept for Severe Steroid-Refractory Acute Graft-versus-Host Disease. Biol Blood Marrow Transplant 22:179–182. https://doi.org/10.1016/j.bbmt.2015.08.039.
5. Van Lint MT, Milone G, Leotta S, et al (2006) Treatment of acute graft-versus-host disease with prednisolone: significant survival advantage for day +5 responders and no advantage for nonresponders receiving anti-thymocyte globulin. Blood 107:4177–4181. https://doi.org/10.1182/blood-2005-12-4851
6. Westin JR, Saliba RM, De Lima M, et al (2011) Steroid-Refractory Acute GvHD: Predictors and Outcomes. Adv Hematol 2011:601953. https://doi.org/10.1155/2011/601953
7. Ruutu T, Gratwohl A, de Witte T, et al (2014) Prophylaxis and treatment of GvHD: EBMT-ELN working group recommendations for a standardized practice. Bone Marrow Transplant 49:168–173. doi:10.1038/bmt.2013.107
8. Garnett C, Apperley J, Pavlu (2013) Treatment and management of graft- versus -host disease: improving response and survival. Ther Adv Hematol 4:366–378. https://doi.org/10.1177/2040620713489842
9. Magenau J, Runaas L, Reddy P (2016) Advances in understanding the pathogenesis of graft-versus-host disease. Br J Haematol 173:190–205. https://doi.org/10.1111/bjh.13959.
10. Jagasia MH, Greinix HT, Arora M, et al (2015) National Institutes of Health Consensus Development Project on Criteria for Clinical Trials in Chronic Graft-versus-Host Disease: I. The 2014 Diagnosis and Staging Working Group report. Biol Blood Marrow Transplant 21:389–401.e1. https://doi.org/10.1016/j.bbmt.2014.12.001
11. Choi J, Ziga ED, Ritchey J, et al (2012) IFNγR signaling mediates alloreactive T-cell trafficking and GvHD. Blood 120:4093–4103. https://doi.org/10.1182/blood-2012-01-403196
12. Ma HH, Ziegler J, Li C, et al (2011) Sequential activation of inflammatory signaling pathways during graft-versus-host disease (GvHD): early role for STAT1 and STAT3. Cell Immunol 268:37–46. https://doi.org/10.1016/j.cellimm.2011.01.008
13. Elli EM, Baratè C, Mendicino F, Palandri F, Palumbo GA (2019) Mechanisms Underlying the Anti-inflammatory and Immunosuppressive Activity of Ruxolitinib. Front Oncol 9:1186. https://doi.org/10.3389/fonc.2019.01186
14. Heine A, Held SA, Daecke SN, et al (2013) The JAK-inhibitor ruxolitinib impairs dendritic cell function in vitro and in vivo. Blood 122:1192–1202. https://doi.org/10.1182/blood-2013-03-484642
15. Zeiser R, von Bubnoff N, Butler J, et al (2020) Ruxolitinib for Glucocorticoid-Refractory Acute Graft-versus-Host Disease. N Engl J Med 382:1800–1810. doi:10.1056/NEJMoa1917635
16. Zeiser R, Polverelli N, Ram R, et al (2021) Ruxolitinib for Glucocorticoid-Refractory Chronic Graft-versus-Host Disease. N Engl J Med 385:228–238. doi:10.1056/NEJMoa2033122
17. Przepiorka D, Luo L, Subramaniam S, et al (2020) FDA Approval Summary: Ruxolitinib for Treatment of Steroid-Refractory Acute Graft-Versus-Host Disease. Oncologist 25:e328–e334. https://doi.org/10.1634/theoncologist.2019-0627.
18. Wolf A, Masow J, Billings M (2022) Novartis receives European Commission approval for Jakavi® to be the first post-steroid treatment for acute and chronic graft-versus-host disease. Publishing Novartis.com. https://www.novartis.com/news/media-releases/novartis-receives-european-commission-approval-jakavi-be-first-post-steroid-treatment-acute-and-chronic-graft-versus-host-disease. Accessed: 05 May 2023.
19. Martini DJ, Chen YB, DeFilipp Z (2022) Recent FDA Approvals in the Treatment of Graft-Versus-Host Disease. Oncologist 27:685–693. https://doi.org/10.1093/oncolo/oyac076
20. Ferrara JL, Harris AC, Greenson JK, et al (2011) Regenerating islet-derived 3-alpha is a biomarker of gastrointestinal graft-versus-host disease. Blood 118:6702–6708. https://doi.org/10.1182/blood-2011-08-375006
21. Weber D, Frauenschläger K, Ghimire S, et al (2017) The association between acute graft-versus-host disease and antimicrobial peptide expression in the gastrointestinal tract after allogeneic stem cell transplantation. PLoS One 12:e0185265. https://doi.org/10.1371/journal.pone.0185265
22. Solán L, Kwon M, Carbonell D, et al (2019) ST2 and REG3α as Predictive Biomarkers After Haploidentical Stem Cell Transplantation Using Post-transplantation High-Dose Cyclophosphamide. Front Immunol 10:2338. https://doi.org/10.3389/fimmu.2019.02338
23. Glucksberg H, Storb R, Fefer A, et al (1974) Clinical manifestations of graft-versus-host disease in human recipients of marrow from HL-A-matched sibling donors. Transplantation 18:295–304. doi:10.1097/00007890-197410000-00001
24. Thomas E, Storb R, Clift RA, et al (1975) Bone-marrow transplantation (first of two parts) N Engl J Med 292:832–843. doi:10.1056/NEJM197504172921605
25. Thomas ED, Storb R, Clift RA, et al (1975) Bone-marrow transplantation (second of two parts) N Engl J Med 292:895–902. doi:10.1056/NEJM197504242921706
26. Przepiorka D, Weisdorf D, Martin P, et al (1995) 1994 Consensus Conference on Acute GvHD Grading. Bone Marrow Transplant 15:825–828.
27. Vander Lugt MT, Braun TM, Hanash S, et al (2013) ST2 as a marker for risk of therapy-resistant graft-versus-host disease and death. N Engl J Med 369:529–539. doi:10.1056/NEJMoa1213299
28. Paczesny S, Krijanovski OI, Braun TM, et al (2009) A biomarker panel for acute graft-versus-host disease. Blood 113:273–278. doi:10.1182/blood-2008-07-167098
29. Srinagesh HK, Özbek U, Kapoor U, et al (2019) The MAGIC algorithm probability is a validated response biomarker of treatment of acute graft-versus-host disease. Blood Adv 3:4034–4042. https://doi.org/10.1182/bloodadvances.2019000791
30. Zeiser R, Blazar BR (2017) Acute Graft-versus-Host Disease - Biologic Process, Prevention, and Therapy. N Engl J Med 377:2167–2179. doi:10.1056/NEJMra1609337
31. Ferrara JL, Levine JE, Reddy P, Holler E (2009) Graft-versus-host disease. Lancet 373:1550–61. https://doi.org/10.1016/S0140-6736(09)60237-3
32. Cahn JY, Klein JP, Lee SJ et al (2005) Prospective evaluation of 2 acute graft-versus-host (GvHD) grading systems: a joint Société Française de Greffe de Moëlle et Thérapie Cellulaire (SFGM-TC), Dana Farber Cancer Institute (DFCI), and International Bone Marrow Transplant Registry (IBMTR) prospective study. Blood 106:1495–1500. https://doi.org/10.1182/blood-2004-11-4557
33. MacMillan ML, Weisdorf DJ, Wagner JE, et al (2002) Response of 443 patients to steroids as primary therapy for acute graft-versus-host disease: comparison of grading systems. Biol Blood Marrow Transplant 8:387–394. https://doi.org/10.1053/bbmt.2002.v8.pm12171485
34. Biavasco F, Ihorst G, Wasch R, et al (2022) Therapy response of glucocorticoid-refractory acute GvHD of the lower intestinal tract. Bone Marrow Transplant 57:1500–1506. https://doi.org/10.1038/s41409-022-01741-3
35. Martin PJ, Rizzo JD, Wingard JR, et al (2012) First- and second-line systemic treatment of acute graft-versus-host disease: recommendations of the American Society of Blood and Marrow Transplantation. Biol Blood Marrow Transplant 18:1150–1163. https://doi.org/10.1016/j.bbmt.2012.04.005
36. Przepiorka D, Luo L, Subramaniam S, et al (2020) FDA Approval Summary: Ruxolitinib for Treatment of Steroid-Refractory Acute Graft-Versus-Host Disease. Oncologist 25:e328-e334. https://doi.org/10.1634/theoncologist.2019-0627
37. Jagasia M, Perales MA, Schroeder MA, et al (2020) Ruxolitinib for the treatment of steroid-refractory acute GvHD (REACH1): a multicenter, open-label phase 2 trial. Blood 135:1739–1749. https://doi.org/10.1182/blood.2020004823
38. Polverelli N, Breccia M, Benevolo G, et al (2017) Risk factors for infections in myelofibrosis: role of disease status and treatment. A multicenter study of 507 patients. Am J Hematol 92:37–41. https://doi.org/10.1002/ajh.24572
39. Dioverti MV, Abu Saleh OM, Tande AJ (2018) Infectious complications in patients on treatment with Ruxolitinib: case report and review of the literature. Infect Dis (Lond) 50:381–387. https://doi.org/10.1128/cmr.00035-19
40. Abedin S, McKenna E, Chhabra S, et al (2019) Efficacy, Toxicity, and Infectious Complications in Ruxolitinib-Treated Patients with Corticosteroid-Refractory Graft-versus-Host Disease after Hematopoietic Cell Transplantation. Biol Blood Marrow Transplant 25:1689–1694. https://doi.org/10.1016/j.bbmt.2019.04.003
41. Khoury HJ, Langston AA, Kota VK, et al (2018) Ruxolitinib: a steroid sparing agent in chronic graft-versus-host disease. Bone Marrow Transplant 53:826–831. https://doi.org/10.1038/s41409-017-0081-5

**Tables and figures**

**Table 1 Clinical characteristics of patients with aGvHD**

| **Characteristics** | **aGvHD**  **n=49** |
| --- | --- |
| Male, n (%) | 29 (59) |
| Female, n (%) | 20 (41) |
| Age, median, (range) (in years) | 55 (46–61) |
| **Diagnosis** | **n (%)** |
| AML | 24 (49) |
| ALL | 5 (10) |
| MPN | 5 (10) |
| MDS | 5 (10) |
| NHL | 4 (8) |
| Hodgkin’s lymphoma | 2 (4) |
| Multiple myeloma | 2 (4) |
| Others (aplastic anemia, pulmonary lymphoid granulomatosis) | 2 (4) |
| **Donor type** | **n (%)** |
| URD | 39 (80) |
| RD | 10 (20) |
| **aGvHD maximal grade before ruxolitinib** | **n (%)** |
| Grade I | 4 (8) |
| Grade II | 19 (39) |
| Grade III | 16 (33) |
| Grade IV | 10 (20) |
| **Severity of aGvHD at start of ruxolitinib** | **n (%)** |
| Grade I | 7 (14) |
| Grade II | 22 (45) |
| Grade III | 15 (31) |
| Grade IV | 5 (10) |
| **Organ manifestation of aGvHD at start of ruxolitinib** | **n (%)** |
| Skin | 18 (37) |
| Gut | 30 (61) |
| Liver | 1 (2) |
| **aGvHD onset, median (range)** | 20 (16–27) |
| Abbreviations: aGvHD=acute graft-versus-host disease; ALL=acute lymphoblastic leukemia; AML=acute myeloid leukemia; MDS=myelodysplastic syndrome; GvHD; MPN=myeloproliferative neoplasia; NHL=non-Hodgkin’s lymphoma; RD=related; URD=unrelated. | |

**Table 2 Ruxolitinib characteristics and concomitant IST**

| **Characteristics** | **Med. (IQR)** |
| --- | --- |
| Start of ruxolitinib after allo-SCT (days) | 59 (41–97) |
| Duration of aGvHD before start of ruxolitinib (days) | 11 (7–20) |
| Duration of ruxolitinib application (median days) | 37 (20–86) |
| Day of end of ruxolitinib after allo-SCT, (days) | 103 (67–185) |
| Duration of follow-up (days) | 501 (95–905) |
| Dose of ruxoltinib at start (mg) | 20 (10–20) |
| **Additional ISM* at the beginning of ruxolitinib** | **n (%)** |
| One ISM | 1 (2) |
| Two ISMs | 23 (47) |
| Three ISMs | 20 (41) |
| Four ISMs | 5 (10) |
| **Most common combinations of ISM** | **n (%)** |
| Prednisolone and CNI | 22 (45) |
| Prednisolone, CNI and Etanercept | 10 (20) |
| Prednisolone, CNI and MMF | 7 (14) |
| Other combinations^a^ | 10 (20) |
| Steroids (mg/kg body weight) at start of ruxolitinib, median (range) | 1.3 (0.6–1.9) |
| **Indication for ruxolitinib** | **n (%)** |
| Steroid-dependent aGvHD | 15 (31) |
| Steroid-refractory aGvHD | 34 (69) |
| **Number of prior therapy lines before ruxolitinib** | **n (%)** |
| One | 33 (67) |
| Two | 11 (22) |
| Three | 3 (6) |
| Four | 2 (4) |
| *Every combination consisted of prednisolone.  ^a^The combinations such as prednisolone, CNI, mycophenolate mofetil (MMF), etanercept, extracorporal photopheresis (ECP), and antithymocyte globulin (ATG) in different combinations.  aGvHD=acute graft-versus-host disease; Allo-SCT=Allogeneic hematopoietic stem cell transplantation; IQR= interquartile range; ISM=Immunosuppressive medication. | |

**Table 3 Safety within 1-month follow-up**

|  | **At start of ruxolitinib AE of any grade, n (%)** | **Within 1 month AE of any grade, n (%)** | **p-value** | **At start of ruxolitinib SAE, n (%)** | **Within 1 month SAE, n (%)** | **p-value** |
| --- | --- | --- | --- | --- | --- | --- |
| **Anemia** | 42 (86) | 42 (86) | 0.999 | 21 (43) | 29 (59) | 0.057 |
| **Thrombocytopenia** | 44 (90) | 49 (100) | 0.063 | 24 (49) | 32 (65) | 0.057 |
| **Neutropenia** | 10 (20) | 19 (39) | 0.035 | 5 (10) | 14 (29) | 0.033 |
| **Bacterial infection – events** | - | 16 |  | - | 4 |  |
| **Mycotic infection – events** | - | 2 |  | - | 2 |  |
| **Viral infection – events** | - | 34 |  | - | 8 |  |
| **Infectious complications per patient** | - | 31 (63) |  | - | 10 (20) |  |
| Abbreviations: AE=adverse event; CTCAE=common terminology criteria for adverse events; SAE=severe adverse event. SAE is defined as ≥ CTCAE Grade III. | | | | | | |

**Table 4 Patient characteristics and** risk factors

|  | **n** | **Responder***  **N=32** | **Non-responder**  **N=17** |
| --- | --- | --- | --- |
|  | | | |
| **Age^#^ (med, IQR)** | 49 | 54 (43-59) | 58 (54–66) |
| **Weight (med, IQR)** | 49 | 82 (61-92) | 80 (72–86) |
| **Gender** | 49 |  |  |
| male (%) | 29 | 17 (59) | 12 (41) |
| female (%) | 20 | 15 (75) | 5 (25) |
| **Time to ruxolitinib (med, IQR)** | 49 | 12 (7–20) | 10 (8–22) |
| **Ruxolitinib dose (med, IQR)** | 49 | 20 (10–20) | 10 (10–20) |
| **Grade aGvHD (med, IQR)** | 49 | 2 (2–3) | 2 (2–4) |
| **Additional ISM (med, IQR)** | 49 | 2 (2–3) | 3 (3-4) |
| **Therapy lines before ruxolitinib (med, IQR)** | 49 | 1 (1–1) | 2 (1–2) |
| **Response to steroids** | 49 |  |  |
| Steroid-refractory (%) | 34 | 18 (53) | 16 (47) |
| Steroid-dependent (%) | 15 | 14 (93) | 1 (7) |
| **Main affected organ site** | 49 |  |  |
| Skin | 18 | 12 | 6 |
| Liver | 1 | 1 | 0 |
| Gut | 30 | 19 | 11 |
| **CTCAE anemia (med, IQR)** | 49 | 1 (1–4) | 4 (1–4) |
| **CTCAE thrombocytopenia (med, IQR)** | 49 | 2 (1–4) | 3 (1–4) |
| **CTCAE neutropenia (med, IQR)** | 49 | 0 (0-0) | 0 (0–2) |
| *defined as CR or PR after one month  ^#^ age at transplantation  Abbreviations: CTCAE=common terminology criteria for adverse events; CR, complete response; IQR= interquartile range; Med=median; PR, partial response | | | |

**Table 5 Factors associated with response**

|  | **OR** | **95% CI** | | **p-value** |
| --- | --- | --- | --- | --- |
| **Age** | 0.95 | 0.90 | 1.00 | 0.067 |
| **Weight** | 1.00 | 0.96 | 1.03 | 0.79 |
| **Sex** | 2.12 | 0.61 | 7.42 | 0.24 |
| **Time to ruxolitinib** | 1.00 | 0.97 | 1.03 | 0.99 |
| **Initial ruxolitinib dose** | 1.086 | 0.97 | 1.22 | 0.17 |
| **Severity of aGvHD** | 0.54 | 0.26 | 1.13 | 0.102 |
| **Additional ISM** | 0.24 | 0.083 | 0.66 | 0.006 |
| **Treatment lines before ruxolitinib** | 0.49 | 0.22 | 1.07 | 0.072 |
| **Affected organ site** | 0.92 | 0.50 | 1.71 | 0.79 |
| **Response to steroids (dependent vs refractory)** | 12.44 | 1.47 | 105.51 | 0.021 |
| **CTCAE anemia** | 0.80 | 0.55 | 1.16 | 0.24 |
| **CTCAE thrombocytopenia** | 0.78 | 0.51 | 1.20 | 0.26 |
| **CTCAE neutropenia** | 0.71 | 0.43 | 1.16 | 0.17 |
| **2BM MAP, start ruxolitinib** | 0.13 | 0.004 | 4.27 | 0.25 |
| Abbreviations: aGvHD=acute graft-versus-host disease; BM=biomarker; CTCAE=common terminology criteria for adverse events; ISM=immunosuppressive medication; MAP= MAGIC Algorithm Probability | | | | |

**Table 6 Serum biomarker levels of Reg3α and ST2 at onset of aGvHD and ruxolitinib**

|  | **Responder*** | **Non-responder** | **p-value** |
| --- | --- | --- | --- |
| **Reg3α (ng/mL), onset GvHD (med, IQR)** | 74 (28–131), n=20 | 94 (39–491), n=10 | 0.33 |
| **Reg3α (ng/mL), start ruxolitinib (med, IQR)** | 66 (37–100), n=23 | 92 (35–400), n=15 | 0.26 |
| **ST2 (pg/mL), onset GvHD (med, IQR)** | 52,349 (30,266–109,892), n=20 | 86,841 (47,691–131,663), n=10 | 0.24 |
| **ST2 (pg/mL), start ruxolitinib (med, IQR)** | 66,877 (33,947–105,776), n=23 | 79,018 (35,930–126,039), n=15 | 0.50 |
| **2BM MAP, onset GvHD (med, IQR)** | 0.22 (0.11–0.31), n=20 | 0.26 (0.18–0.49), n=10 | 0.27 |
| **2BM MAP, start ruxolitinib (med, IQR)** | 0.24 (0.15–0.34), n=23 | 0.25 (0.16–0.43), n=15 | 0.36 |
| **2BM Ann Arbor Score (AA1+AA2), onset GvHD (n, %)** | 14 (74) | 5 (26) | 0.28 |
| **2BM Ann Arbor Score (AA3), onset GvHD (n, %)** | 6 (55) | 5 (45) |
| **2BM Ann Arbor Score (AA1+AA2), start ruxolitinib (n, %)** | 14 (64) | 8 (36) | 0.65 |
| **2BM Ann Arbor Score (AA3), start ruxolitinib (n, %)** | 9 (56) | 7 (44) |
| *defined as CR or PR after one month Abbreviations: aGvHD=acute graft-versus-host disease; BM=biomarker; IQR=interquartile range; Med=median; MAP=MAGIC Algorithm Probability; ST=suppression of tumorigenicity. | | | |

**Fig. 1 Response assessment.**  ORR and FFS over time after initiation of ruxolitinib therapy: ORR and FFS are shown in percentage of all patients included at the respective time point.

**
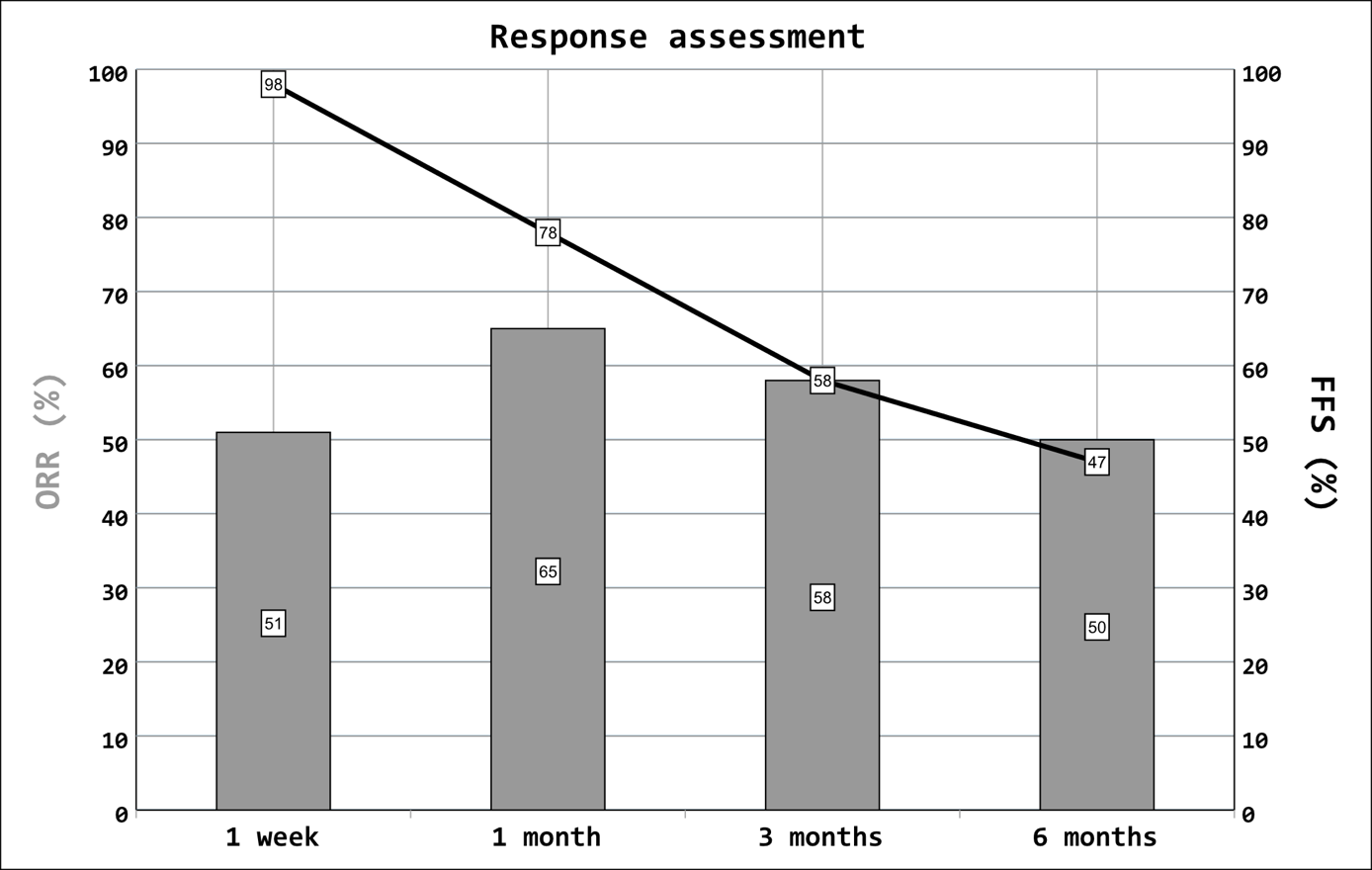
**

FFS=failure free survival; ORR=overall response rates.

**Fig. 2 Steroid taper during follow-up.** Steroid dose per kg bodyweight within a 6-month follow-up period. A significant reduction is already observed from the first week of treatment onwards (* = P value ≤0.001, treatment-induced changes are analyzed with Wilcoxon test, data are presented as median with interquartile range). A direct comparison of the steroid dose between the 1-week and 1-month follow-up also displayed a significant decrease. Patients receiving steroids due to cGvHD were excluded from this calculation.

RUX=ruxolitinib.


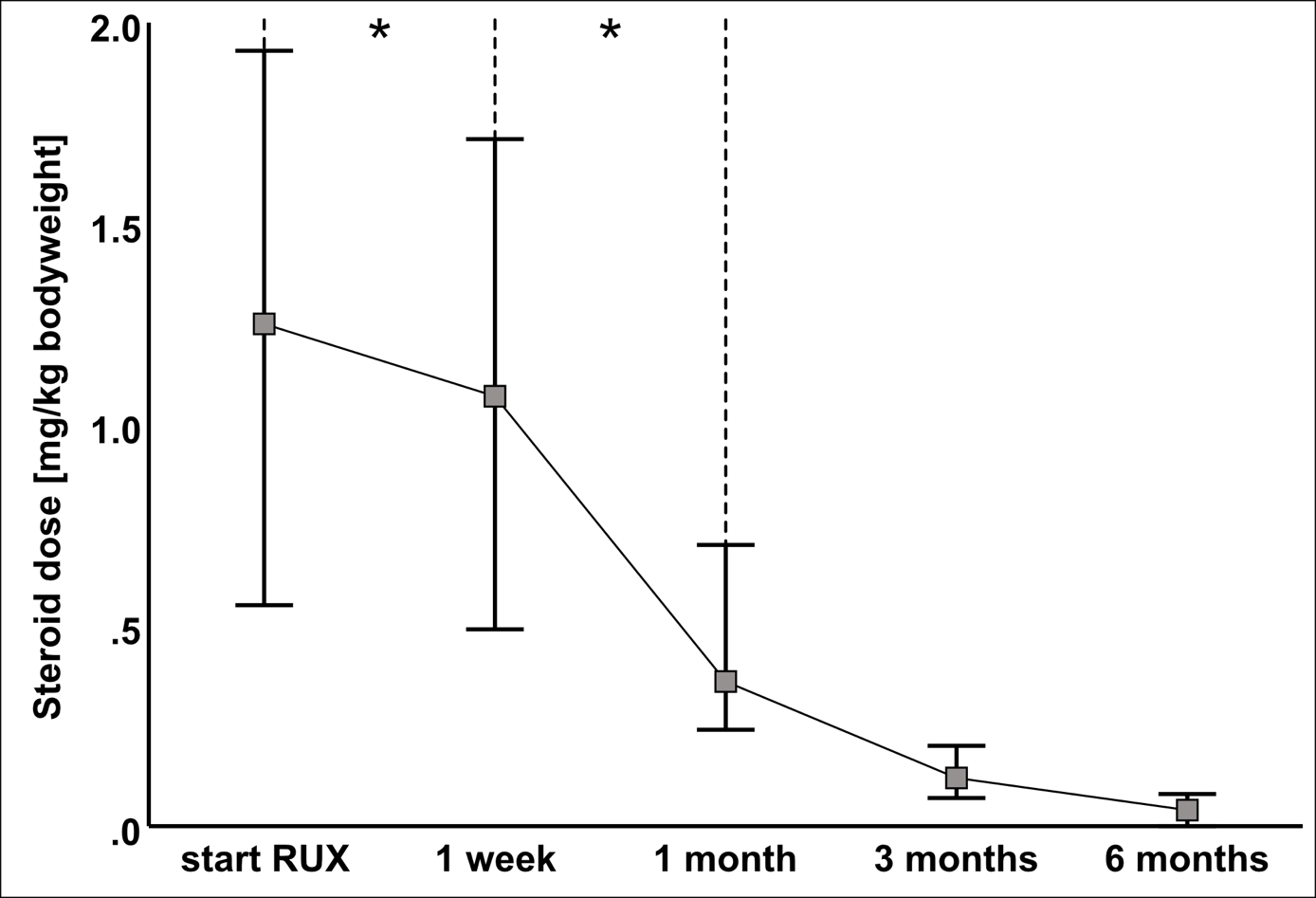


**Fig. 3 Assessment of FFS depending on MAP score.** FFS over the time depending on MAP score (MAP score 1+2 vs MAP score 3). Patients with higher MAP showed poorer FFS. FFS is shown in percentage. 38 patients are included in this analysis (with 22 patients MAP 1+2 and 16 patients MAP 3).


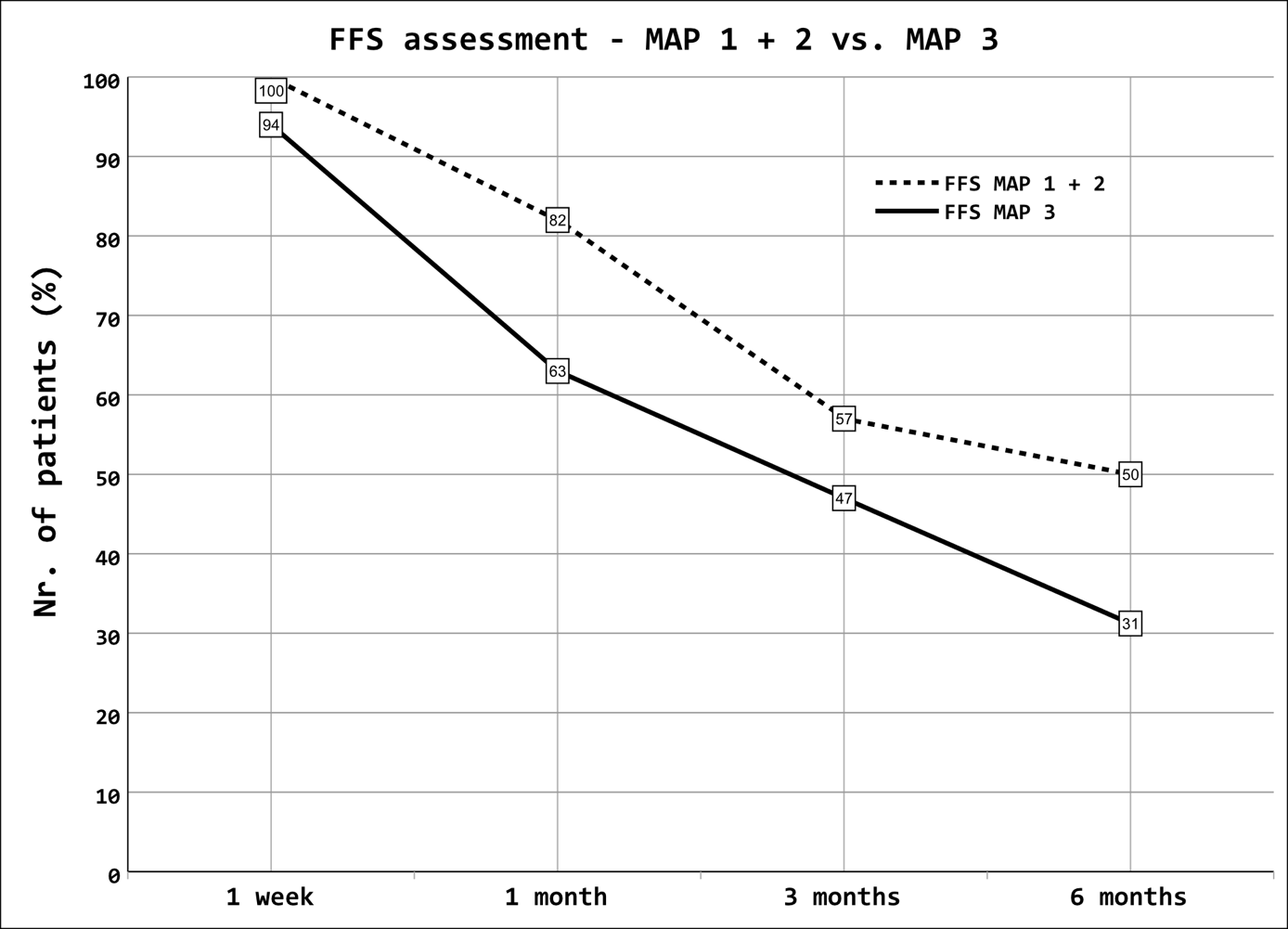


FFS=failure free survival; MAP= MAGIC Algorithm Probability
